# Comparison of Influenza Epidemic Trends Based on a Large-scale Claims Database and National Infectious Disease Surveillance in Japan

**DOI:** 10.2188/jea.JE20250565

**Published:** 2026-07-05

**Authors:** Takenori Yamauchi, Hiroki Den, Shouhei Takeuchi, Masaya Saito, Mitsuo Uchida, Akatsuki Kokaze

**Affiliations:** 1 Department of Hygiene, Public Health and Preventive Medicine, School of Medicine, Showa Medical University, Tokyo, Japan; 2 Department of Nutrition Science, Faculty of Nursing and Nutrition, University of Nagasaki, Nagasaki, Japan; 3 Department of Information Security, Faculty of Information Systems, University of Nagasaki, Nagasaki, Japan; 4 Gunma University Center for Mathematics and Data Science, Maebashi, Japan

**Keywords:** influenza, claim database, epidemiology, mathematical model

## Abstract

**Background:**

Seasonal influenza is a recurrent respiratory infection, and timely detection is essential for public health. In Japan, surveillance is conducted through sentinel medical institutions under the National Epidemiological Surveillance of Infectious Diseases (NESID). Recently, access to large claims databases, such as the JMDC claims database (JMDCdb), has increased. While both are sample-based systems, JMDCdb covers a much larger population. We aimed to assess consistency between these sources in estimating influenza cases and the effective reproduction number (*R_t_*) and to explore their utility in epidemic analysis.

**Methods:**

We analyzed data from week 36 of 2016 to week 35 of 2019. Influenza cases were estimated from NESID (reported cases and cases per sentinel) and JMDCdb (cases with influenza-related diagnoses and antiviral prescriptions). Daily infection counts were derived to estimate *R_t_*.

**Results:**

Although minor differences appeared at epidemic peaks, estimates from NESID reports aligned well with JMDCdb. Estimates based on cases per sentinel were lower. *R_t_* values were consistent across data sources. *R_t_* exceeded 1.0 when cases per sentinel surpassed 0.2–0.3. Using a threshold of 0.25 cases per sentinel enabled detection of epidemic onset 4–5 weeks earlier than current standards.

**Conclusion:**

Claims data, such as those from JMDCdb, may be useful for retrospective examination of influenza trends. Moreover, a detailed analysis of the number of cases reported per sentinel suggested the potential to propose threshold values that enable earlier prediction of epidemics than conventional criteria.

## INTRODUCTION

Seasonal influenza (hereafter, influenza) is a respiratory infectious disease caused by influenza viruses and occurs annually during the winter season. In Japan, under the Infectious Diseases Control Law enacted in 1999, influenza has been designated as a notifiable disease in the National Epidemiological Surveillance of Infectious Diseases (NESID). Approximately 5,000 sentinel medical institutions are required to report the number of cases weekly to the public health centers (PHCs).^1^ These data are aggregated at the PHC level and eventually compiled for all 47 prefectures, with the results published on the website of the National Institute of Infectious Diseases (NIID).^2^ Administrators of sentinel medical institutions are required to report cases every Monday when a physician diagnoses or suspects influenza based on characteristic clinical symptoms or in the event of a death. Even when not all of the four typical symptoms—sudden onset, high fever, upper respiratory inflammation, and general fatigue or systemic symptoms—are observed, reporting is also required if viral antigens are detected with rapid diagnostic kits using nasopharyngeal aspirates, nasal swabs, or throat swabs. Separate reporting criteria are defined for core sentinel sites.^3^

As influenza poses a threat to public health, the issuance of advisories and alerts helps to prevent potential outbreaks. An advisory indicates a possibility of a large-scale outbreak within the next 4 weeks, while an alert is issued when such an outbreak is ongoing or suspected. These advisories and alerts are issued at the PHC level and contribute to early detection of spread and timely warning within PHC jurisdictions. Specifically, an advisory is triggered when the number of cases per sentinel exceeds 10 in a week, and an alert when it exceeds 30. However, if an alert was already issued in the previous week, it continues as long as the number of cases per sentinel remains above 10. These thresholds are based on specific probabilities; for example, the alert threshold is designed so that a series of alerts during an outbreak would occur with a probability of approximately 1% over the past 5 years.^4^ Recently, some local governments have explicitly stated on their websites that an epidemic period is considered to begin when the number of cases per sentinel exceeds 1.0.^5^^–^^8^ The effective reproduction number (*R_t_*) is defined as the average number of secondary infections generated by a single case in a given population.^9^ When *R_t_* exceeds 1.0, the number of infections is expected to increase; when it falls below 1.0, infections are expected to decline. Therefore, *R_t_* is considered a useful indicator of the transmissibility of infectious diseases.^10^ However, an *R_t_* value exceeding 1.0 does not necessarily indicate the onset of an epidemic, as local clusters or stochastic extinction may prevent the transition to a sustained outbreak. Nevertheless, when based on data aggregated at the national level and when the lower limit of the 95% credible interval of *R_t_* continuously exceeds 1.0, this metric can be interpreted as a strong signal of epidemic expansion. Therefore, although *R_t_* is not sufficient for determining the occurrence of an epidemic, it can serve as a supplementary quantitative indicator for identifying the expansion phase of transmission. Our previous analysis using data from Miyazaki Prefecture confirmed that *R_t_* exceeded 1.0 at the time advisories were issued, indicating that advisories were announced during the expansion phase of infection.^11^

Meanwhile, the criteria for selecting sentinel sites are defined in the NESID implementation guidelines. For example, in the case of pediatric sentinel sites, prefectural governments select from among institutions that offer pediatric care (primarily pediatric clinics). The number of sentinel sites is determined based on the population within each PHC jurisdiction: one site for populations up to 30,000, two sites for populations of 30,000 to 75,000, and so on. Thus, for diseases such as influenza, which are subject to sentinel surveillance, the system operates as a form of epidemiological sampling. In recent years, studies using health insurance claims data have become widespread. The JMDC claims database (JMDCdb), provided by JMDC Inc., covers approximately 10 million people, mainly employees of large companies and their dependents, out of 29 million members of health insurance societies. As of April 1, 2024, Japan’s total population was about 124 million, implying that the JMDCdb represents roughly 1/12^th^ of the national population.

In this study, we examine the consistency between NESID, a sample-based surveillance system, and JMDCdb, which is also a sample but of a far larger scale. To enable direct comparison across the three data sources, we expressed all measures as estimated total case numbers, converting “cases per sentinel” into total cases using population-based scaling. We first compared the estimated number of influenza cases based on NESID reports and sentinel data with those derived from JMDCdb. Then, we compared the effective reproduction numbers estimated from each data source. Finally, we evaluated retrospectively how epidemic status inferred from *R_t_* relates to existing surveillance thresholds.

## METHODS

### Data sources and case definition

We used influenza data published by NIID^2^ and calculated both the weekly number of reported influenza cases and the weekly number of cases per sentinel from this source. As the number of reported influenza cases drastically declined during the coronavirus disease 2019 pandemic and potential confounding effects could not be ruled out, we limited the analysis to three influenza seasons from week 36 of 2016 to week 35 of 2019.

In JMDCdb, we defined influenza cases as those with both an influenza-related diagnosis and a prescription for anti-influenza medication. This definition was adopted for two reasons. First, in Japan, the standard clinical approach to influenza emphasizes early antiviral treatment for all patients to shorten the fever duration and prevent severe outcomes, including death.^12^^,^^13^ Consequently, a high proportion of patients diagnosed with influenza receive antivirals, with one report estimating the prescription rate at 85%.^14^ Second, this case definition ensures high specificity. In Japan, a diagnosis must be recorded when medical procedures are performed. For example, influenza antibody testing requires an influenza-related diagnostic code regardless of the test result. In principle, a “suspected” flag is attached in the claims data; but in practice, this is not always the case. As antiviral medications should be prescribed within 48 hours of symptom onset, patients with positive tests but no prescriptions would not be included under our case definition. Conversely, defining cases based on diagnostic codes alone would likely lead to overestimation. Therefore, while underestimation cannot be ruled out, we defined cases based on the presence of both an influenza-related diagnosis and a prescription for anti-influenza medication.

From JMDCdb, we extracted the following variables:

● From the subscriber table: date of birth, sex, relationship to insured, start and end of observation.
● From the diagnosis table: visit date, standardized disease name, and date of diagnosis.
● From the drug table: visit date, drug name, prescription date, and dispensing date.
● From the medical procedure table: visit date, standardized procedure name, and implementation date.

Dates for birth, observation period, and visit were unified by setting the day as the first of each month (eg, a birth month of March 2015 was treated as March 1, 2015). Definitions for influenza-related disease names, drug names, and standardized procedures are provided in Table 1, Table 2, and Table 3, respectively. As reporting date was a key element in our analysis, we defined it as follows:

**Table 1.** Standard disease names associated with seasonal influenza cases

Name of Influenza	Japanese Standard Disease Code	ICD-10 Codes
Influenza (H5N1)	8848119	J09-
Influenza encephalomyelitis	8830730	J118
Influenza-associated sinusitis	8830727	J111
Influenza A Soviet strain	8842079	J101
Influenza-associated otitis media	3810006	J118
Influenza myelitis	8830728	J118
Avian influenza	8843940	J101
Avian influenza (H7N9)	8847722	J101
Influenza B	8842082	J101
Influenza-associated bronchitis	8830710	J111
Influenza	4871001	J111
Influenza A	8842080	J101
Influenza-associated acute upper respiratory tract infection	8830723	J111
Influenza-associated laryngitis	8830725	J111
Influenza (H1N1) 2009	8846356	J09-
Influenza pneumonia	8830731	J110
Influenza myocarditis	8830720	J118
Influenza-associated pharyngitis	8830722	J111
Acute influenza myocarditis	8832283	J118
Influenza encephalopathy	8843828	J118
Influenza-associated gastroenteritis	8830721	J118
Influenza A Hong Kong strain	8842081	J101
Influenza-associated laryngotracheitis	8830726	J111

ICD, International Statistical Classification of Diseases, 10^th^ Revision.

**Table 2.** Standard medicine names associated with seasonal influenza cases

Medicine Name	Medicine Code	Active Ingredient
Inavir Inhalation Powder, details unknown	100000067130	Laninamivir Octanoate Hydrate
Tamiflu Dry Syrup, details unknown	100000028745	Oseltamivir Phosphate
Tamiflu Capsules, details unknown	100000028708	Oseltamivir Phosphate
Oseltamivir Phosphate Capsules, manufacturer unknown	100000044506	Oseltamivir Phosphate
Oseltamivir Phosphate for Syrup, manufacturer unknown	100000044507	Oseltamivir Phosphate
Rapiacta Intravenous Infusion Vial, details unknown	100000069927	Peramivir Hydrate
Xofluza Tablets, details unknown	100000082653	Baloxavir, Marboxil
Rapiacta Intravenous Infusion Bag, details unknown	100000069928	Peramivir Hydrate
Oseltamivir Capsules “Sawai,” details unknown	100000083346	Oseltamivir Phosphate
Inavir Inhalation Powder 20 mg	100000067131	Laninamivir Octanoate Hydrate
Tamiflu Dry Syrup 3%	100000002779	Oseltamivir Phosphate
Xofluza Tablets 20 mg	100000082652	Baloxavir, Marboxil
Relenza	100000012390	Zanamivir Hydrate
Oseltamivir DS 3% “Sawai”	100000083064	Oseltamivir Phosphate
Tamiflu Capsules 75	100000002295	Oseltamivir Phosphate
Oseltamivir Capsules 75 mg “Sawai”	100000083053	Oseltamivir Phosphate
Rapiacta Intravenous Infusion Bag 300 mg	100000069929	Peramivir Hydrate
Xofluza Tablets 10 mg	100000082651	Baloxavir, Marboxil
Rapiacta Intravenous Infusion Vial 150 mg	100000069930	Peramivir Hydrate
Relenza, details unknown	100000031673	Zanamivir Hydrate
Inavir Inhalation Suspension 160 mg Set	100000084831	Laninamivir Octanoate Hydrate

**Table 3.** Standard medical procedure names associated with seasonal influenza cases

Standardized Medical Procedure Code	Standardized Medical Procedure Name
160042210	Influenza Virus A Antibody Titer (Qualitative, Semi-Quantitative, Quantitative)
160042310	Influenza Virus B Antibody Titer
160169450	Influenza Virus Antigen Precision Test
160198010	Influenza Nucleic Acid Detection
160224750	SARS-CoV-2 & Influenza Nucleic Acid Simultaneous Detection (Outsourced Testing)
160224850	SARS-CoV-2 & Influenza Nucleic Acid Simultaneous Detection (Non-Outsourced Testing)
160226450	SARS-CoV-2 & Influenza Virus Antigen Simultaneous Detection
160229650	SARS-CoV-2 & Influenza Nucleic Acid Simultaneous Detection (Outsourced Testing)
160229750	SARS-CoV-2 & Influenza Nucleic Acid Simultaneous Detection (Non-Outsourced Testing)
160230050	SARS-CoV-2 & Influenza Virus Antigen Simultaneous Detection (Qualitative)
160235250	SARS-CoV-2, Influenza & RSV Nucleic Acid Simultaneous Detection (Outsourced Testing)
160235350	SARS-CoV-2, Influenza & RSV Nucleic Acid Simultaneous Detection (Non-Outsourced Testing)
160235450	SARS-CoV-2, Influenza & RSV Antigen Simultaneous Detection (Qualitative)
160235650	Influenza Virus Infection Test (Endoscopic Telescope Use)
999900673	[J] SARS-CoV-2 & Influenza Nucleic Acid Simultaneous Detection (Non-Outsourced Testing)
999900674	[J] SARS-CoV-2 & Influenza Nucleic Acid Simultaneous Detection (Outsourced Testing)

RSV, respiratory syncytial virus; SARS-CoV-2, severe acute respiratory syndrome coronavirus 2.

If any of the diagnosis start date, prescription date, or dispensing date occurred within 32 days of the visit date, the earliest of them was treated as the reporting date. If none of these met the criterion, but a valid procedure date was available and within 32 days of the visit date, the procedure date was used. Cases not satisfying either condition were excluded. When individuals had multiple reporting dates within the same visit month, we retained both if they were at least 14 days apart; otherwise, only the earlier date was used. We then aggregated the number of reported cases by reporting date. Age at report was estimated using reporting date and date of birth. Monthly population denominators were calculated from the observation start and end dates.

### Estimation of total number of influenza cases

In this study, we estimated the total number of influenza-related medical visits in Japan using three different methods:

1. Using the number of reported cases from NESID;
2. Using the number of cases per sentinel from NESID;
3. Using data from JMDCdb.

In all cases, when the estimated number of cases included decimal values, we rounded to the nearest whole number greater than or equal to zero.

#### 1. Estimation using the number of reported cases from NESID

According to NIID, the estimated number of patients during the following periods was 10,460,000 (from week 36 of 2016 to week 20 of 2017), 14,580,000 (from week 36 of 2017 to week 17 of 2018), and 12,005,000 (from week 36 of 2018 to week 17 of 2019).^15^^,^^16^ In this study, these estimates are treated as the total number of infected individuals, rather than merely those who sought medical care. Let *T_j_* denote the number of reported cases in week *j*, and let $\hat{I_{k}}$ denote the estimated number of infections for the aggregation period *k*. The correction coefficient *c_k_* was estimated using Equation (1), and the weekly number of infections $\hat{I_{j}}$ was then estimated using Equation (2):

$$\hat{I_{k}}=\sum_{j}c_{k}\times T_{j},$$ (1)

$$\hat{I_{j}}=c_{k}\times T_{j}.$$ (2)

#### 2. Estimation using the number of cases per sentinel from NESID

We directly used the values of “cases per sentinel” published by NESID. In addition, the weekly number of sentinel sites was back-calculated from the publicly available “number of reported cases” and “cases per sentinel.” Using this estimated number of sentinel sites and the monthly population data, we calculated the population per sentinel. Finally, the weekly number of infections was estimated by multiplying the “cases per sentinel” by the “population per sentinel.” For weeks that spanned 2 calendar months, we used the population of the month containing the greater number of days. For example, week 35 of 2016 (August 29 to September 4) includes 3 days in August and 4 days in September; therefore, we used the September population figure for this week. This method was applied to evaluate the impact of differences in sentinel site distribution and population composition on the estimation of infection counts, in comparison with the nationally adjusted estimate based on reported cases (method 1).

#### 3. Estimation using JMDCdb

Using the number of reported cases per day and the monthly number of subscribers in JMDCdb, we estimated the number of daily reports per 100,000 population. We then applied Japan’s monthly population estimates, published by the Statistics Bureau of the Ministry of Internal Affairs and Communications, to estimate the total number of reported cases nationwide per day. These daily values were aggregated by week to obtain weekly estimates of the number of infections.

### Estimation of the effective reproduction number

As estimation of *R_t_* requires daily case counts, we converted weekly case counts obtained in methods 1 and 2 into daily counts using the same approach as in our previous study.^11^ Specifically, for each season, we constructed a cumulative incidence curve, applied a smoothing spline to reduce random fluctuations, and then calculated the day-to-day differences in the cumulative values to derive the estimated number of daily infections $\hat{I_{t}}$ . When using JMDCdb, we directly used the available daily infection counts as $\hat{I_{t}}$ . We assumed that the effective reproduction number *R_j_* remains constant within each week and estimated it using the following equation:

$$\hat{E}(\hat{I_{t}})=R_{j}\sum_{\tau =1}^{T}\omega_{\tau }\hat{I_{t-\tau }},$$ (3)

where *ω_τ_* represents the serial interval distribution for *τ* days before day *t*. In this study, we assumed a shifted gamma distribution^17^ with a mean of 2.6 days and a standard deviation of 1.5 days^18^^,^^19^ as the serial interval distribution. Each parameter was estimated using Markov Chain Monte Carlo (MCMC) under the assumption that the estimated daily number of infections $\hat{I_{t}}$ follows a Poisson distribution with a mean $\hat{E}(\hat{I_{t}})$ . The MCMC settings were as follows: warm-up = 5,000, iterations = 20,000, number of chains = 4, and thinning interval = 10. Convergence of the chains was assessed using the Gelman–Rubin statistic (R-hat), with values below 1.1 considered indicative of convergence.

### Determination of epidemic onset threshold

In this study, the onset of an epidemic was defined as the condition wherein the lower limit of the 95% credible interval of *R_j_* continuously exceeded 1.0. Based on this criterion, threshold values of the number of cases per sentinel were compared using 0.1, 0.2, 0.25, and 0.3 as candidates, including the range of 0.1–0.2 reported in a previous study,^20^ to examine a reasonable range for epidemic detection.

### Statistical analysis and software

This study was conducted using anonymized data from JMDCdb. All personally identifiable information had been completely removed prior to data provision, and no individual could be identified from the data. According to the “Ethical Guidelines for Life Science and Medical Research Involving Human Subjects” established by the Ministry of Health, Labour and Welfare, studies using anonymized secondary data are not subject to ethical review. Therefore, approval by an institutional review board and informed consent were not required. In this study, data were extracted from JMDCdb using PostgreSQL version 16, operated from a Windows 11 (Microsoft Corp., Redmond, WA, USA) terminal. All numerical analyses were conducted using Microsoft Excel 2019 (Microsoft Corp.) or R version 4.3.2 (R Foundation for Statistical Computing, Vienna, Austria). For the MCMC procedures, we used Stan version 2.32.2 called from within R.

## RESULTS

The demographic characteristics of influenza cases obtained from JMDCdb are presented in Table 4. Among males, the number of primary insured individuals and their dependents was approximately equal, whereas among females, the number of dependents was more than three times that of primary insured individuals. In terms of age at infection, the 0–9- and 10–19-year age groups were most frequently affected in both sexes, followed by those in their 40s. However, when examining the proportion of infected individuals among those covered by health insurance, the 0–9- and 10–19-year age groups remained the most affected, but the next most affected group was 20–29 years old. Overall, the infection rate tended to decline with increasing age.

**Table 4.** Background information on seasonal influenza cases

	Male	Female
Principal/Family		
Principal	632,725	256,646
Family	672,572	870,828

Relationship		
Principal	612,850	251,947
Partner	1,128	262,203
Children	647,525	575,024
Other	5,170	9,842
Unknown	38,624	28,458

Age, years		
	20 (9–41)	19 (9–41)

Age class, years, *n* (%)		
0–9	347,577 (26.9)	316,616 (23.2)
10–19	292,611 (22.1)	292,611 (20.5)
20–29	144,480 (12.4)	144,480 (8.6)
30–39	158,983 (10.6)	158,983 (8.4)
40–49	184,350 (9.6)	184,350 (8.2)
50–59	131,522 (10.0)	131,522 (7.6)
60–69	41,449 (7.6)	41,449 (5.7)
70–79	4,011 (4.5)	3,923 (4.2)

Age was presented as the median (25th–75th percentile). The proportion of influenza cases among the total number of subscribers on October 1st for the years 2016, 2017, and 2018 was presented by age group, expressed as a percentage.

**Figure 1.**
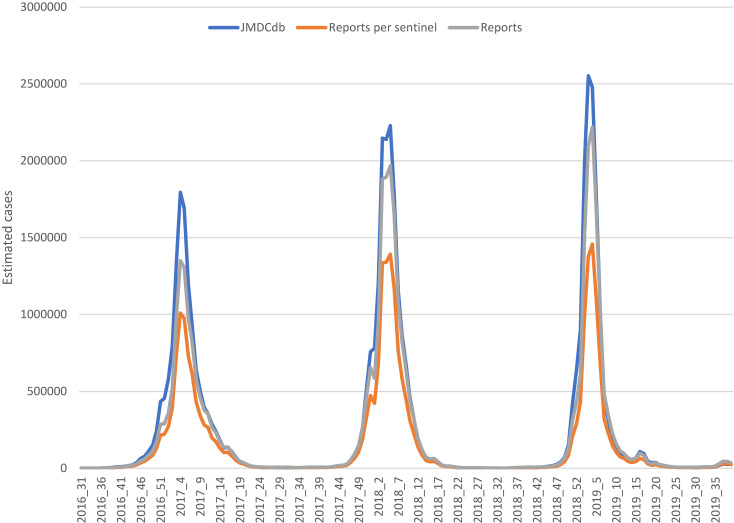
Estimated seasonal influenza cases based on three datasets. “JMDCdb,” “Reports per sentinel,” and “Reports” represent estimated seasonal influenza cases based on JMDCdb, number of reports per sentinel, and number of total reports, respectively. On the horizontal axis, the first four digits indicate the year, and the digits following the underscore represent the epidemiological week.

Figure 1 shows the estimated number of seasonal influenza cases based on JMDCdb, the number of cases per sentinel, and the number of reported cases. In order of magnitude, the data sources ranked as JMDCdb, reported cases, and cases per sentinel. Two noteworthy points were observed. First, although the estimate provided by the National Institute of Infectious Diseases (NIIDest) was based on the number of reported cases, estimates based on the number of cases per sentinel were consistently lower than NIIDest in every week. Second, while the estimated peak case count from JMDCdb exceeded NIIDest during the 2017/18 and 2018/19 seasons, estimates from both sources were well aligned during the non-peak periods of those seasons. In particular, strong agreement was observed in the early stages of each season.

**Figure 2.**
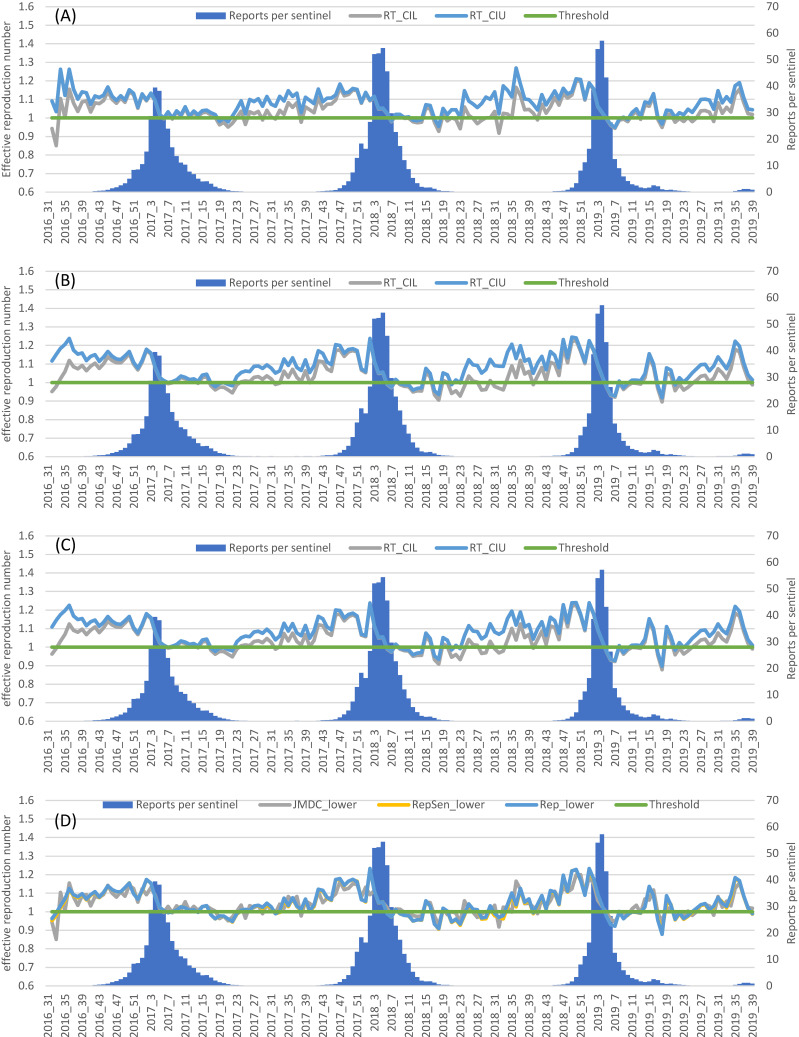
Estimated effective reproduction number based on three datasets. “Reports per sentinel” refers to the number of cases reported per sentinel site and is shown as bar graphs. “RT_CIL” and “RT_CIU” represent the lower and upper bounds of the 95% credible interval of the estimated effective reproduction number, respectively. “Threshold” indicates the critical value of the effective reproduction number (1.0). In panel (d), “JMDC_lower,” “RepSen_lower,” and “Rep_lower” represent the lower bounds of the 95% credible intervals of the effective reproduction number estimated using JMDCdb, reports per sentinel, and total reports, respectively. Results based on different data sources used to estimate the effective reproduction number: (**A**) JMDCdb, (**B**) reports per sentinel, and (**C**) total reports. (**D**) compares the lower bounds of the 95% credible intervals across the three data sources.

Figure 2 presents the weekly estimates of *R_j_*. The peak weeks for the 2016/2017, 2017/2018, and 2018/2019 seasons were week 4 of 2017, week 5 of 2018, and week 4 of 2019, respectively. When using JMDCdb, the lower bound of the 95% credible interval for *R_j_* consistently exceeded 1.0 starting from week 34 of 2016, week 39 of 2017, and week 42 of 2018 (Figure 2A). Using the number of cases per sentinel, the corresponding weeks were week 34 of 2016, week 41 of 2017, and week 41 of 2018 (Figure 2B). When the number of reported cases was used, the lower limit of the 95% credible interval of *R_j_* continuously exceeded 1.0 in week 34 of 2016, week 33 of 2017, and week 41 of 2018 (Figure 2C). Figure 2D shows only the lower bounds of the 95% credible intervals of *R_j_* estimated from the three data sources. The *R_j_* values estimated using the number of cases per sentinel and reported cases were nearly identical. While *R_j_* based on JMDCdb exhibited slightly different behavior, it largely agreed with the other two estimates during periods of expanding transmission.

**Figure 3.**
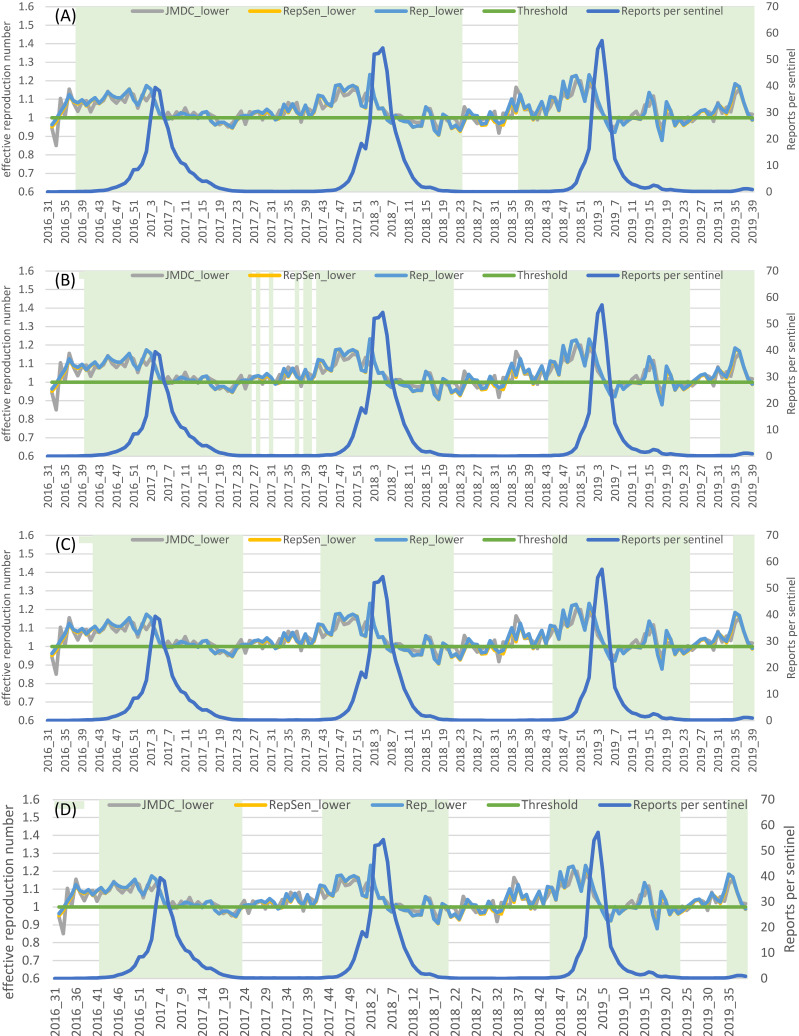
Estimated effective reproduction number and the timing for determining an epidemic. “JMDC_lower,” “RepSen_lower,” and “Rep_lower” represent the lower bounds of the 95% credible intervals of the effective reproduction number estimated using JMDCdb, reports per sentinel, and total reports, respectively. The shaded green area indicates the period during which the pandemic occurred. In (**A**), (**B**), (**C**), and (**D**), the thresholds for determining an epidemic based on reports per sentinel are set to 0.1, 0.2, 0.25, and 0.3, respectively.

Figure 3A presents the epidemic periods assuming a threshold of 0.1 for the number of cases per sentinel. Some weeks fell within the epidemic period even when the lower bound of the effective reproduction number’s 95% credible interval was below 1.0. Furthermore, no non-epidemic period was observed between the 2016/2017 and 2017/2018 seasons. Figure 3B shows the epidemic periods assuming a threshold of 0.2. A distinctive pattern appeared in 2017, where multiple short epidemic periods emerged, clearly indicating that this threshold is insufficient for long-term epidemic forecasting. Figure 3C and Figure 3D show the results for thresholds of 0.25 and 0.3, respectively. In both cases, the weeks judged to mark the beginning of an epidemic were week 42 of 2016, week 43 of 2017, and week 45 of 2018. Notably, from these weeks onward, the *R_j_* values estimated from all data sources consistently exceeded 1.0, supporting the consistency between epidemic classification and *R_j_* dynamics.

## DISCUSSION

This study examined the consistency of seasonal influenza epidemic trends derived from different data sources (NESID and JMDCdb), providing new insights into estimating the effective reproduction number and establishing a threshold for cases per sentinel as an indicator of outbreaks. A particularly noteworthy finding was that, although the estimated number of infections at the peak was slightly higher when using JMDCdb than when using NESID-reported cases, the estimates from NESID and JMDCdb showed good agreement. Furthermore, the effective reproduction numbers estimated from both NESID and JMDCdb were largely consistent. In addition, based on the trends in the effective reproduction number, it was inferred that an outbreak was likely underway when the number of cases per sentinel exceeded 0.3.

JMDCdb used in this study is an epidemiological claims database that compiles inpatient, outpatient, and pharmacy claims, as well as health checkup data, submitted by multiple health insurance societies. Therefore, the data primarily cover workers and their families and do not include data for older adults. As shown in Table 4, there were no enrollees aged ≥80 years during the study period. Noda et al estimated the number of seasonal influenza patients using the National Database of Health Insurance Claims and Specific Health Checkups of Japan (NDB), which comprehensively covers medical data nationwide.^21^ Although differences in case definitions prevent direct comparison of absolute numbers, age-specific infection rates can be compared. By re-aggregating Noda’s results and dividing the number of cases in each age group for the periods from September 2017 to August 2018 and from September 2018 to August 2019 by the population as of October 1 of each year, we calculated age-specific infection rates. Likewise, from JMDCdb, we obtained age-specific infection rates by dividing the number of infected individuals by the number of enrollees as of October 1 for the corresponding periods. As shown in eTable 1, there were no substantial differences in age-specific infection rates. This likely explains why the infection count estimates based on JMDCdb exceeded those from NIID in Figure 1—that is, the incidence rate of influenza is generally lower among older adults than among younger individuals. Consequently, in a dataset that excludes older adults, the overall average incidence rate tends to be relatively higher, resulting in an overestimation of the total number of infections compared with the national estimates. However, the number of JMDCdb enrollees increased from approximately 5.3 million in August 2016 to approximately 9.3 million in September 2019, suggesting that the estimation accuracy improved over time. This improvement is considered to reflect not only the expansion of the sample size but also the increased regional and occupational diversity among younger and middle-aged populations, thereby enhancing the representativeness of nationwide infection trends. Moreover, because older adults generally have lower infection rates and contribute relatively little to the total number of infections except during epidemic peaks, it is likely that the improvement in estimation accuracy associated with the increase in enrollees outweighed the impact of excluding older adults during non-peak periods. Next, the estimated number of infections based on the number of cases per sentinel was considerably lower than that based on the number of reported cases. During the study period, each sentinel facility covered approximately 25,000 people on average, but in reality, this coverage varied, which may explain part of the discrepancy. For example, the estimated population of Tokyo in February 2025 was 14,190,090,^22^ and the number of sentinel facilities was 419,^23^ implying each sentinel covered about 33,867 people. In contrast, in Miyazaki Prefecture in week 7 of 2025, the number of reported cases and the number of cases per sentinel were 210 and 3.62, respectively,^24^ suggesting that there were 58 sentinel facilities. Given Miyazaki’s estimated population of 1,026,874 in February 2025,^25^ each sentinel covered approximately 17,704 people. This indicates that the distribution and coverage of sentinel sites vary by region. Additionally, differences in facility size and medical specialties (eg, whether a pediatric sentinel offers only pediatric care or also internal medicine and surgery) likely lead to variations in patient volume and demographics. For these reasons, infection estimates based on cases per sentinel may tend to be underestimated. Furthermore, as estimates using cases per sentinel become roughly equivalent to those using reported cases when multiplied by 1.4, it suggests that each sentinel covers approximately 35,000 people on average nationwide.

In this study, we estimated the effective reproduction number using the serial interval of influenza A. According to a report by NIID, the A(H3) subtype was dominant during the 2016/2017 season, and the proportion of influenza B detections increased approximately week 9 of 2017.^26^ Similarly, in the 2018/2019 season, the A(H1pdm09) subtype was predominant, with a notable increase in A(H3) cases in the latter half of the season. Although influenza B was detected toward the end of the season, its case numbers remained low.^16^ In contrast, during the 2017/2018 season, the overall proportions of A(H1pdm09), A(H3), and B were 24%, 30%, and 46%, respectively.^27^ As the serial interval of influenza B is reported to be approximately 3.7 days,^28^ the accuracy of estimating the effective reproduction number may be limited during periods with high numbers of influenza B cases. However, in the 2016/2017 and 2018/2019 seasons, almost no influenza B cases were detected until week 2 of 2017 and week 2 of 2019, respectively, suggesting that influenza A was predominant until just before the epidemic peak. Moreover, in the 2017/2018 season, detections of influenza A were more frequent until approximately week 51 of 2017. Given that this study assumed a weekly constant value for the effective reproduction number, the impact of differences in serial intervals on its estimation is likely limited. That being said, the observed discrepancy in the estimated timing of the epidemic onset during the 2017/2018 season when using reported case data—as opposed to other data sources—might be partially attributable to changes in the dominant influenza virus type.

This study examined the relationship between the effective reproduction number and the number of cases per sentinel to assess epidemic onset. The findings suggest that the threshold for determining epidemic onset may lie between 0.2 and 0.3 cases per sentinel. Yamamoto et al analyzed 10 years of data using an epidemic trend indicator; they found that in the Hyogo Prefecture, even if the threshold was set at 0.1 to 0.2 cases per sentinel, once exceeded, the number of cases rarely declined and instead proceeded to a full-scale epidemic.^20^ Although their methodology and study period differed from ours, the fact that our results are largely consistent with theirs is noteworthy. Furthermore, Yamamoto et al reported that using a threshold of 0.1 cases per sentinel allowed epidemic onset to be detected approximately 1 month earlier than the conventional threshold of 1.0 case per sentinel.^20^ In our study, using a threshold of 0.23 enabled us to detect the onset of the epidemic 5 weeks earlier in both the 2016/2017 and 2017/2018 seasons, and 4 weeks earlier in the 2018/2019 season. As mentioned earlier, because sentinel placement and population coverage vary by region, it is necessary to establish region-specific thresholds using locally accumulated data. Nonetheless, our findings suggest that a detailed analysis of cases per sentinel could enable influenza outbreaks to be forecasted approximately 1 month in advance. Early identification of the onset of an epidemic enables healthcare systems to proactively prepare resources—strengthening outpatient capacity, securing hospital beds, adjusting staff schedules, and ensuring adequate supplies of antiviral drugs and protective equipment—potentially preventing system overload during peak periods. It also facilitates targeted interventions in high-risk settings; local governments can issue timely alerts to schools, childcare facilities, and elderly care homes to reinforce infection control measures, while healthcare providers can advise vulnerable populations (elderly patients, immunocompromised patients, pregnant women, and chronically ill patients) to take increased precautions before community transmission intensifies. In addition, advance warnings enable public health authorities to launch timely awareness campaigns promoting preventive behaviors, such as hand hygiene and respiratory etiquette, which may be more effective when initiated before widespread transmission. Therefore, the lower threshold approach demonstrated in this study could enable gradual, timely responses that can help mitigate the impact of an epidemic and reduce burdens on healthcare systems and communities.

However, the practical implementation of new thresholds in infectious disease surveillance requires comparability across regions and consistency in administrative decision-making. The current criterion of 1.0 case per sentinel has been used for many years based on long-standing operational experience, and abrupt changes could cause confusion. In addition, application of a lower threshold might increase the risk of false alarms in regions where local transmission has not yet been established, potentially affecting the credibility of public health alerts. Therefore, application of newly proposed thresholds, such as those suggested by Yamamoto et al and supported by this study, would require verification of reproducibility across multiple years and regions, as well as consensus building among public health authorities. These processes increase the feasibility of integrating scientifically grounded thresholds into routine surveillance practice.

This study has a few limitations. First, regarding JMDCdb, the age distribution it covers is limited. However, as discussed earlier, we believe the impact of this limitation on our study is minimal. Second, another limitation is the definition of infected individuals when using JMDCdb. As a claims database, JMDCdb does not provide test results, even if influenza testing was performed. Thus, identifying influenza cases relies on diagnostic codes and prescribed medications. In this study, we defined influenza cases as those with an influenza-related diagnosis and a prescription for anti-influenza medication. In addition to previously cited reports, other studies have reported that 85–90% of influenza patients in Japan are prescribed antiviral drugs.^29^ While the possibility of underestimating the number of cases cannot be ruled out, the impact on our findings is likely limited. Even if the prescription rate were slightly lower, as long as the rate remained relatively constant throughout the season, it would not affect the estimation of the effective reproduction number—again suggesting minimal impact.

The findings of this study suggest that JMDCdb has strong representativeness for diseases such as seasonal influenza, in which infections among the elderly are relatively uncommon. However, because it does not include information on the residential locations of enrollees, it is not suitable for detailed regional analyses. Furthermore, as claims data are generated by medical institutions on a monthly basis and require additional processing by insurers before being incorporated into the database, their use for real-time infectious disease surveillance is not feasible in practice. This study aimed to retrospectively analyze claims data to assess and complement the validity of existing infectious disease surveillance indicators, such as those provided by NESID. In the future, by utilizing data sources such as NESID or NDB, which allow for region-level analyses, it may be possible to establish more precise thresholds for epidemic onset and to contribute to the improvement of infectious disease surveillance systems.

## SUPPLEMENTARY MATERIAL

eTable 1. Age-specific infection rate
