# Supplementary material for: Comparison of Influenza Epidemic Trends Based on a Large-scale Claims Database and National Infectious Disease Surveillance in Japan
Source: J Epidemiol. 2026 Jul 5;36(7):223–32. doi: 10.2188/jea.JE20250565 (PMC13265304; doi:10.2188/jea.JE20250565)
Supplement: Supplementary file 1 [file je-36-223-s001.pdf]

eTable 1. Age-specific infection rate

Age class, years	September, 2017–August, 2018		September, 2018–August, 2019	
	JMDCdb	Noda et al.	JMDCdb	Noda et al.
0–9	29.4	31.3	23.0	24.9
10–19	22.4	22.4	16.9	16.8
20–29	7.9	8.3	9.7	9.6
30–39	8.8	9.2	9.7	9.7
40–49	9.7	9.6	8.4	8.2
50–59	8.7	8.3	7.1	6.8
60–69	6.8	5.9	5.6	4.9
70–79	5.0	4.4	4.1	3.7
≥80	-	4.9	-	4.1

JMDCdb, JMDC claims database.
